# Associations Between Maternal Anxiety and Depression During Pregnancy and Obstetric Outcomes: A Cross-Sectional Study

**DOI:** 10.7759/cureus.86541

**Published:** 2025-06-22

**Authors:** Dipika Vidhan, Jitendra Rohilla, Vishal Dhiman, Kavita Khoiwal

**Affiliations:** 1 Psychiatry, Centre for Mental Health and Counselling-Nepal, Kathmandu, NPL; 2 Psychiatry, All India Institute of Medical Sciences, Rishikesh, Rishikesh, IND; 3 Obstetrics and Gynecology, All India Institute of Medical Sciences, Rishikesh, Rishikesh, IND

**Keywords:** anxiety, depression, maternal social support, obstetric outcome, partner support, pregnancy

## Abstract

Background

Anxiety and depressive symptoms are common during pregnancy, influencing obstetric outcomes. This cross-sectional study primarily aimed to assess the prevalence of anxiety and depressive symptoms in pregnancy and their association with obstetric outcomes. Another objective of the study was to determine the association of maternity social support and partner support during pregnancy with the final obstetric outcomes.

Methods

During the first phase (antenatal) of the study, anxiety, depressive symptoms, maternity social support, and partner support were assessed using structures and validated questionnaires, i.e., Generalized Anxiety Disorder scale (GAD-7), Edinburgh Postnatal Depression Scale, Maternity Social Support Scale, and Partner Support Scale, respectively. During phase II (post-partum), details on the mode of delivery, gender of the baby, gestational age at delivery, birth weight, and any complication(s) during delivery were noted.

Results

Out of a total of 104 study participants, anxiety and depressive symptoms were present in 17.3% (n=18) and 26.0% (n=27), respectively. The risk of low birth weight was increased with clinically significant antenatal anxiety symptoms (χ2 = 7.566, p=0.010) and depressive symptoms (χ2=4.323, p=0.038). A strong association was found between fetal maturity and maternity social support (χ2=26.269, p≤0.001).

Conclusions

The study results highlight the importance of routine mental health screening during pregnancy and timely interventions to improve maternal and neonatal outcomes.

## Introduction

Pregnancy is one of a woman's most significant life events, generally perceived as a period of joy and anticipation. However, it also brings substantial social, psychological, and physiological changes that can result in various mental and emotional challenges. Among these, psychological distress is particularly common, with anxiety affecting 18.2-24.6%, followed by depression in 10-15% of pregnant women [1-5]. These mental health issues can have detrimental effects on maternal and fetal outcomes, influencing fetal development, labor process, delivery outcomes, and post-partum adjustment [6-9]. Risk factors for antenatal depression include financial instability, previous girl child, perceiving the need for a boy child, previous pregnancy complications, stressful life events, relationship problems, low educational status, and marital violence [3,10]. Conversely, strong family and partner support have been shown to act as protective factors in shaping pregnancy experience [11,12]. If left unaddressed, antenatal anxiety and depression can lead to unfavorable obstetric outcomes such as preterm delivery, stillbirth, and impaired fetal growth [3,9].

While extensive research has been conducted on the physical aspects of pregnancy, the psychological dimension remains relatively under-explored, particularly in low- and middle-income countries (LMICs) where socio-cultural and systemic challenges may amplify mental health burdens [1,9]. Much of the existing evidence originates from high-income countries and may not reflect the unique stressors, support systems, and health outcomes relevant in developing settings. The emotional health of pregnant women remains one of the most neglected components of obstetric care in these regions, despite its critical importance [9].

This study aims to address this gap by exploring the prevalence of anxiety and depressive symptoms among pregnant women and examining their associations with obstetric outcomes in a developing country context. Additionally, it investigates how maternity social support and partner support influence these outcomes. The study is planned to support the growing body of evidence on maternal mental health by providing region-specific data that are currently scarce. Unlike previous studies that predominantly focus on Western populations, this research highlights culturally relevant determinants and protective factors in pregnancy-related mental health in a developing country setting. This study adds to the culturally relevant data from India, where traditional family dynamics and expectations may influence maternal mental health.

By contextualizing psychological health within the local socio-cultural fabric, this study offers insights that may not only inform clinical practice but also guide targeted interventions and public health strategies. Improving screening, support systems, and interventions can reduce perinatal complications, promote maternal well-being, and enhance early childhood development contributing to broader public health goals, including maternal and child health improvement and healthcare system strengthening.

## Materials and methods

This was a hospital-based, cross-sectional observational study (with follow-up data collection) to determine anxiety, depression, partner support, and maternity social support during pregnancy and their association with various obstetric outcomes. Pregnant women in their third trimester, at a gestational age of 24-36 weeks, availing antenatal service from the outpatient department (OPD) of Obstetrics and Gynecology, and between 18 and 35 years of age with a low-risk singleton pregnancy were screened for recruitment in the study. Those having a history of bipolar disorder and/or psychotic disorder, having child delivery outside the index study center, and not being able to read and write were excluded from the study.

The permission for the study was obtained from the local Institutional Ethics Committee, and the study was conducted from December 2022 to May 2023. Those satisfying the inclusion and exclusion criteria were explained the rationale of the study, and written informed consent was taken. A total of 133 participants who fulfilled the requirements were approached using a purposive sampling technique. The data were collected in two phases: phase I (during the 24-36 weeks of gestation period) and phase II (after childbirth, i.e., post-partum phase).

In the first phase, a pre-designed semi-structured proforma was applied to collect socio-demographic and obstetric details of the subjects. Participants completed the self-reported scales to assess depressive symptoms, anxiety, maternity social support (family support, friendship network, help from partner, conflict with spouse, feeling controlled by partner, and feeling unloved by partner), and partner support (emotional and financial) during pregnancy using the Edinburgh Postnatal Depression Scale (EPDS) [13], Generalized Anxiety Disorder scale (GAD-7) [14], Maternity Social Support Scale (MSSS) [15], and Partner Support Scale (PSS) [16,17], respectively.

EPDS is a 10-item self-administered questionnaire, and each item is scored on a 4-point scale from 0 to 3. The score ranges from 0 to 30, with a cut-off of 13 for depression, and the higher the scores, the more severe the depression [13]. The GAD-7 (7-item) self-rating scale was developed to measure the severity of anxiety. Each item has a score between 0 and 3, and the total score runs from 0 to 21, with scores ranging from 0 to 4 indicating minimal anxiety, 5-9 indicating mild anxiety, 10-14 indicating moderate anxiety, and 15-21 indicating severe anxiety. Higher scores correspond to more severe anxiety symptoms [14]. MSSS, a 6-item, self-reported, 5-point Likert scale created for perinatal depression, has a maximum possible score of 30, with higher numbers denoting greater support: 0-18 (low support), 19-24 (medium support), and >24 (adequate) [15]. On the other hand, PSS is an 8-item self-reported scale with scores ranging from 8 to 32. Partner’s support is classified into supportive and unsupportive based on the median scale score. It has excellent dependability with Cronbach’s α of 0.95 [16]. GAD-7 and EPDS are in the public domain and free to use. However, due permissions for the use of MSSS and PSS were obtained from the original authors.

Responses were discussed with participants and their family members for re-confirmation to enhance the reliability and validity of the data collected, particularly given the sensitive nature of mental health assessments during pregnancy. Based on the responses, appropriate cut-off scores were applied to assess any depressive or anxiety symptoms. Moderately and severely symptomatic participants were advised to visit the Psychiatry OPD for further assessment and needful.

During the second phase, for those participants who delivered at our center, details of the baby, mode of delivery, gender, gestational age at delivery, birth weight, and any complications during delivery were collected from the medical records.

Out of the 133 eligible participants recruited in the first phase, 24 delivered outside the center, four could not complete the assessment, and one met with intrauterine fetal death; hence, a total of 29 participants were excluded from the analyses.

The collected data were entered into Microsoft Excel (Microsoft Corp., Redmond, WA) and then analyzed using the SPSS Statistics trial version 29.0 (IBM Corp., Armonk, NY). The descriptive statistics were computed. The continuous data were examined using the Kolmogorov-Smirnov test for the distribution of normalcy. Mean and standard deviation was used to report continuous data and proportions for the categorical variables. The categorical data were analyzed using either the chi-square test or Fisher's exact test, depending on the percentage of the total number of cells that had an expected count of less than 5. Further, a non-parametric test, the Wilcoxon-Mann-Whitney U test, was used to evaluate the association of continuous data such as age and PSS scores with other categorical variables, as the data were not normally distributed.

## Results

Table 1 demonstrates the sociodemographic and obstetric details of the participants (n=104). The mean age of the participants was 26.89±3.40 years (mean±SD). Psychological assessments showed that 26% (n=27) of them experienced depressive symptoms, with an average EPDS score of 9.07 ± 5.20, and 18 (17.3%) had anxiety symptoms, with an average GAD-7 score of 5.78 ± 4. 69. Maternity social support was largely adequate (73.1%) with an average MSSS score of 26.375 ± 3.46, and an average PSS score of 28.57±3.68. An almost equal number of participants had normal vaginal deliveries and lower segment cesarean sections (LSCSs). The majority of babies (81.7%) were delivered at term, i.e., 37 completed weeks of gestation, and 30 (28.8%) had low birth weight (LBW) (<2,500 g). Furthermore, 26% of cases experienced complications during delivery such as pre-eclampsia, gestational hypertension, premature rupture of membrane, meconium-stained liquor, intrauterine growth retardation, and fetal distress. Some of these complications were the indication for emergency LSCS, and the major indication for elective LSCS was a history of cesarean delivery.

**Table 1:** Sample characteristics IQR, interquartile range; LBW, low birth weight; LSCS, lower segment cesarean section delivery; NBW, normal birth weight; NVD, normal vaginal delivery; PSS, Partner Support Scale; SD, standard deviation

Parameter		Value
Age, (in years), mean ± SD / median (IQR) / min.-max.		26.89 ± 3.40 / 27.00 (25.00-29.00) / 19.00-34.00
Age groups, n (%)	18-25 years	36 (34.6%)
	26-30 years	54 (51.9%)
	31-35 years	14 (13.5%)
Religion, n (%)	Hindu	94 (90.4%)
	Muslim	10 (9.6%)
Educational status, n (%)	Primary	1 (1.0%)
	Secondary	16 (15.4%)
	Higher secondary	22 (21.2%)
	Graduate	49 (47.1%)
	Post-graduate	16 (15.4%)
Employment, n (%)	Employed	30 (28.8%)
	Unemployed	74 (71.2%)
Type of family, n (%)	Nuclear	47 (45.2%)
	Joint	57 (54.8%)
Parity, n (%)	Primi-para	66 (63.5%)
	Multi-para	38 (36.5%)
Partner violence		1 (0.96%)
Pregnancy (wanted), n (%)		104 (100.0%)
Past abortion or stillbirth, n (%)		19 (18.3%)
Depression, n (%)		27 (26.0%)
Anxiety, n (%)		18 (17.3%)
Maternity social support, n (%)	Low	3 (2.9%)
	Moderate	25 (24.0%)
	Adequate	76 (73.1%)
PSS score, mean ± SD / median (IQR) / min.-max.		28.57 ± 3.68 / 29.50 (27.00-32.00) / 15.00 - 32.00
Obstetric outcomes		
Mode of delivery, n (%)	NVD	50 (48.1%)
	LSCS	54 (51.9%)
Fetal maturity at birth, n (%)	Preterm	15 (14.4%)
	Term	85 (81.7%)
	Post-term	4 (3.8%)
Gender of baby, n (%)	Male	55 (52.9%)
	Female	49 (47.1%)
Birth weight, n (%)	NBW	74 (71.2%)
	LBW	30 (28.8%)
Complications during delivery, n (%)		27 (26.0%)

Table 2 illustrates the association of antenatal anxiety and depressive symptoms with socio-demographic and obstetric parameters among the subjects. The results suggest that those with poor maternity social support and partner support during pregnancy experienced more depressive as well as anxiety symptoms.

**Table 2:** Association of antenatal anxiety and depressive symptoms with sociodemographic and obstetric factors *p-value significant at <0.05. †Wilcoxon rank-sum test. ‡Chi-square test. PSS, Partner Support Scale; SD, standard deviation

Parameter	Depression present (n=27)	Depression absent (n=77)	Test statistic value	p-value	Anxiety present (n=18)	Anxiety absent (n=86)	Test statistic value	p-Value
Age (years), mean±SD	27.63 ± 3.68	26.64 ± 3.28	1.23^†^	0.45	27.17 ± 4.41	26.84 ± 3.18	687.50^†^	0.45
Age group								
18-25 years	7 (25.9%)	29 (37.7%)	1.58^‡^	0.45	6 (33.3%)	30 (34.9%)	0.19^‡^	0.90
26-30 years	15 (55.6%)	39 (50.6%)			9 (50.0%)	45 (52.3%)		
31-35 years	5 (18.5%)	9 (11.7%)			3 (16.7%)	11 (12.8%)		
Religion								
Hindu	23 (85.2%)	71 (92.2%)	1.13^‡^	0.28	14 (77.8%)	80 (93.0%)	3.98^‡^	0.06
Muslim	4 (14.8%)	6 (7.8%)			4 (22.2%)	6 (7.0%)		
Educational status								
Primary education	0 (0.0%)	1 (1.3%)	2.56^‡^	0.64	0 (0.0%)	1 (1.2%)	4.58^‡^	0.33
Secondary education	3 (11.1%)	13 (16.9%)			3 (16.7%)	13 (15.1%)		
Higher secondary	7 (25.9%)	15 (19.5%)			5 (27.8%)	17 (19.8%)		
Graduation	11 (40.7%)	38 (49.4%)			5 (27.8%)	44 (51.2%)		
Post-graduate	6 (22.2%)	10 (13.0%)			5 (27.8%)	11 (12.8%)		
Employment								
Employed	11 (40.7%)	19 (24.7%)	2.51^‡^	0.11	5 (27.8%)	25 (29.1%)	0.01^‡^	1.00
Unemployed	16 (59.3%)	58 (75.3%)			13 (72.2%)	61 (70.9%)		
Type of family								
Nuclear family	15 (55.6%)	32 (41.6%)	1.58^‡^	0.21	11 (61.1%)	36 (41.9%)	2.22^‡^	0.19
Joint family	12 (44.4%)	45 (58.4%)			7 (38.9%)	50 (58.1%)		
Parity								
Primipara	21 (77.8%)	45 (58.4%)	3.23^‡^	0.07	16 (88.9%)	50 (58.1%)	6.06^‡^	0.01*
Multipara	6 (22.2%)	32 (41.6%)			2 (11.1%)	36 (41.9%)		
Psychosocial and obstetric risk factors								
Pressure to have a baby (yes)	0 (0.0%)	0 (0.0%)	1.23^‡^	1.00	0 (0.0%)	0 (0.0%)	0.03^‡^	1.00
Partner violence (yes)	1 (3.7%)	0 (0.0%)	1.14^‡^	0.26	1 (5.6%)	0 (0.0%)	0.04^‡^	0.17
Pregnancy (wanted)	27 (100.0%)	77 (100.0%)	1.34^‡^	1.00	18 (100.0%)	86 (100.0%)	0.23^‡^	1.00
Past abortion or stillbirth (yes)	4 (14.8%)	15 (19.5%)	0.29^‡^	0.77	6 (33.3%)	13 (15.1%)	3.30^‡^	0.09
Mental illness in family (yes)	0 (0.0%)	0 (0.0%)	0.32^‡^	1.00	0 (0.0%)	0 (0.0%)	0.23^‡^	1.00
Anxiety (present)	11 (40.7%)	75 (97.4%)	44.84^‡^	<0.001*	-	-	-	-
Depression (present)	-	-	-	-	16 (88.9%)	11 (12.8%)	44.84^‡^	<0.001*
Maternity social support								
Low	2 (7.4%)	1 (1.3%)	19.54^‡^	<0.001*	2 (11.1%)	1 (1.2%)	18.80^‡^	<0.001*
Moderate	14 (51.9%)	11 (14.3%)			10 (55.6%)	15 (17.4%)		
Adequate	11 (40.7%)	65 (84.4%)			6 (33.3%)	70 (81.4%)		
PSS score	27.04 ± 4.47	29.10 ± 3.23	738.50^†^	0.02*	26.44 ± 4.91	29.01 ± 3.23	694.00^†^	0.02*

Table 3 depicts the association of obstetric outcomes to antenatal anxiety, depression, and support levels among the subjects. A significant association was found between participants experiencing depressive symptoms and the likelihood of giving birth to an LBW baby (χ2=4.323, p=0.038), similarly those with anxiety symptoms (χ2=7.566, p=0.010). A highly significant p-value (χ2=26.269, p≤0.001) suggests a strong association between fetal maturity and levels of perceived social support. Those mothers who perceived low maternity social support during pregnancy were more likely to deliver preterm babies (p<0.001).

**Table 3:** Association of obstetric outcomes with antenatal anxiety, depression, and support levels *p-value significant at <0.05. †Wilcoxon rank-sum test. ‡Chi-square test. LBW, low birth weight; LSCS, lower segment cesarean section delivery; NBW, normal birth weight; NVD, normal vaginal delivery; PSS, Partner Support Scale

Parameter	Mode of delivery		Test statistic value	p-Value	Fetal maturity at birth			Test statistic value	p-Value	Birth weight		Test statistic value	p-Value	Complication during delivery		Test statistic value	p-Value	
	NVD (n=50)	LSCS (n=54)																
					Preterm (n=15)	Term (n=85)	Post-term (n=4)			NBW (n=74)	LBW (n=30)			Yes (n=27)	No (n=77)			
Depression	13 (26.0%)	14 (25.9%)	0.000^‡^	0.993	7 (46.7%)	19 (22.4%)	1 (25.0%)	3.92^‡^	0.135	15 (20.3%)	12 (40.0%)	4.32^‡^	0.038	5 (18.5%)	22 (28.6%)	1.05^‡^	0.305	
Anxiety	39 (78.0%)	47 (87.0%)	1.481^‡^	0.301	10 (66.7%)	73 (85.9%)	3 (75.0%)	3.46^‡^	0.177	66 (89.2%)	20 (66.7%)	7.56^‡^	0.010	22 (81.5%)	64 (83.1%)	0.03^‡^	1.000	
Maternity social support																		
Low	1 (2.0%)	2 (3.7%)	0.43^‡^	0.929	3 (20.0%)	0 (0.0%)	0 (0.0%)	26.27^‡^	<0.001*	1 (1.4%)	2 (6.7%)	4.59^‡^	0.078	0 (0.0%)	3 (3.9%)	2.56^‡^	0.368	
Moderate	13 (26.0%)	12 (22.2%)			7 (46.7%)	18 (21.2%)	0 (0.0%)			15 (20.3%)	10 (33.3%)							
														9 (33.3%)	16 (20.8%)			
Adequate	36 (72.0%)	40 (74.1%)			5 (33.3%)	67 (78.8%)	4 (100.0%)			58 (78.4%)	18 (60.0%)							
														18 (66.7%)	58 (75.3%)			
PSS	28.62 ± 3.82	28.52 ± 3.59	1392.00^†^	0.785	26.40 ± 4.58	28.92 ± 3.41	29.25 ± 3.77	5.98^†^	0.05*	28.97 ± 3.02	27.57 ± 4.87	1245.00^†^	0.329	27.70 ± 4.07	28.87 ± 3.51	847.00^†^	0.149	

There was no association of anxiety or depressive symptoms as well as anxiety and depressive symptoms with obstetric outcomes, as demonstrated in Table 4.

**Table 4:** Association between obstetric outcomes and participants with antenatal depression or anxiety, or both conditions †Wilcoxon rank-sum test. ‡Chi-square test. LBW, low birth weight; LSCS, lower segment cesarean section delivery; NBW, normal birth weight; NVD, normal vaginal delivery; PSS, Partner Support Scale

Parameters	Depression or anxiety		Test statistic value	p-Value	Depression and anxiety		Test statistic value	p-Value
	Present (n = 29)	Absent (n = 75)			Present (n = 11)	Absent (n = 93)		
Mode of delivery								
NVD	15 (51.7%)	35 (46.7%)	2.20^‡^	0.66	9 (56.2%)	41 (46.6%)	0.50^‡^	0.58
LSCS	14 (48.3%)	40 (53.3%)			7 (43.8%)	47 (53.4%)		
Fetal maturity at birth								
Preterm	8(27.6%)	7 (93%)	5.65^‡^	0.06	4 (25.0%)	11 (12.5%)	2.14^‡^	0.34
Term	20 (69.0%)	65 (86.7%)			11 (68.8%)	74 (84.1%)		
Post-term	1 (3.4%)	3 (4.0%)			1 (6.2%)	3 (3.4%)		
Birth weight								
NBW	15 (51.7%)	59 (78.7%)	7.34^‡^	0.09	8 (50.0%)	66 (75.0%)	4.12^‡^	0.07
LBW	14 (48.3%)	16(21.3%)			8 (50.0%)	22 (25.0%)		
Complications during delivery (yes)	7 (24.1%)	20 (26.7%)	0.70^‡^	1.00	3 (18.8%)	24 (27.27%)	0.51^‡^	0.55

## Discussion

The present study showed that 26% had depressive symptoms and 17.3% had anxiety symptoms in their third trimester. Statistical analysis suggested that these symptoms were significantly associated with LBW and that the lower perceived social support during pregnancy was associated with preterm delivery.

Our finding of the prevalence rates of depression is similar to the findings of a systematic review by Gelaye et al. [6] from various LMICs, showing a prevalence of 25.3% (95% CI, 21.4-29.6%), an Indian cross-sectional study by Dahiya et al. [2] reporting 21% prevalence, and the study by Kantipudi et al. [5] demonstrating 22% prevalence. Nevertheless, studies conducted in India revealed a lower rate of prenatal depression, which could be due to the recruitment of participants in the different trimesters and the use of screening and confirmatory tools [3,10,18]. A 17.3% prevalence rate of anxiety in the current study is similar to a survey by Kantipudi et al. [5] from India. However, a higher prevalence was found in a study by Nath et al., which could be because of different study settings, inclusion criteria, and screening tools [19].

Earlier studies have found an association of depression with prior abortion, pressure to have a male child, conflict between partners [3], and other factors such as educational status, employment, and parity [2,10,18]. It is evident that there is an association between antenatal depression and socioeconomic status and alcohol use in partners [2].

We have found an association between maternity social support and partner support with antenatal anxiety and depressive symptoms. A systematic review by Insan et al. [20] from South Asian countries showed a strong association of partner violence and poor relations with in-laws with antenatal depression/anxiety. Also, our finding is in line with a cross-sectional study by Nath et al. [19] that found a significant association between antenatal anxiety and lower social support.

Our finding of an association between antenatal depression and LBW aligns with a meta-analysis by Grote et al. [21] that showed a higher association between LBW and prenatal depression in developing nations (RR=2.05; 95%CI, 1.43-2.93) and a systematic review by Jarde et al. [22] that showed significant risk for neonates, preterm birth, and LBW in those not receiving treatment for depression. A study from Southern India found a significant association of antenatal depression alone and in joint contribution with anxiety and intimate partner violence with an infant's birth weight [23].

An observational cohort study contradicted our findings and found no correlation between LBW and antenatal depression alone but a significant correlation with the combined effect of anxiety and depression, which could be due to factors such as a larger sample size (n=2002), assessment in early pregnancy, and different screening tools [7].

We did find a statistically significant association between anxiety and LBW but no relation with preterm birth and complications during delivery, which is similar to the findings of a meta-analysis by Grigoriadis et al. [24], who demonstrated that anxiety had a positive correlation with preterm birth (pooled OR=1.54; 95% CI, 1.39-1.70) and LBW (OR=1.80; 95% CI, 1.48-2.18). A similar result was reported by Ding et al. [25] (pooled RR=1.76, 95% CI, 1.32-2.33). However, its finding of a positive correlation between anxiety and preterm birth was different from ours. This contrary result could be attributed to differences in assessment timing and study settings.

Our study's finding of a positive correlation between anxiety symptoms and LBW is similar to the finding of an Indian study by Chandra et al. [23] and also the correlation of joint contributions of antenatal anxiety and depression with the birth weight of the baby.

We investigated the association of partner support, maternity social support, and physical violence from partners with adverse obstetric outcomes. There are very few studies to evaluate this relationship. We found a significant association between partner support and maternal anxiety and depressive symptoms but no association with obstetric outcomes. This was similar to the studies by Ilska and Przybyła-Basista [11] and Bloch et al. [26]. No such association was observed while assessing the relationship between social support during pregnancy and obstetric outcome as another secondary objective. Still, we found a substantial association between social support and anxiety and depressive symptoms. This is in line with a study by Paredes et al. [27], who showed that LBW was 3.59 times more likely to occur with poor social support (OR=4.59; 95% CI, 2.27-9.27; P<0.01). A similar study also demonstrated a correlation between low social support during pregnancy and anxiety; however, it did not measure its relationship with obstetric outcomes [19].

Our study has several strengths. We have used depression and anxiety as a clinical condition by employing them as a categorical measure. In a single study, we have examined the association of multiple obstetric outcomes, such as mode of delivery, birth weight, fetal maturity, complications during delivery, and their composite outcome. The tools used were self-administered questionnaires, and participants' responses were re-confirmed by discussing with the participants and family members.

In our study, the smaller sample size may have affected the power of the study. As a cross-sectional study, the long-term relations between study variables could not be studied. Screening tools were used, and diagnostic tools may be used in future studies. Furthermore, the sampling method might potentially limit the generalizability of findings. Additionally, the study did not specifically assess the physical health parameters of participants, which may also play a role in maternal mental health and obstetric outcomes.

## Conclusions

The study highlights the strong correlations of maternal mental health, maternity social support, and partner support with obstetric outcomes. The higher prevalence of antenatal anxiety and depression and its impact on an infant's health warrants the need for regular screening and early intervention of such problems to encourage the health of the mother and for favorable birth outcomes. There must be an integration of comprehensive mental health screening with the national antenatal care programs.
